# Individual Genomic Distinctness of Rice Germplasm as Measured with an Average Pairwise Dissimilarity of Genome-Wide SNPs and Structural Variants

**DOI:** 10.3390/plants14243750

**Published:** 2025-12-09

**Authors:** Yong-Bi Fu

**Affiliations:** Plant Gene Resources of Canada, Saskatoon Research and Development Centre, Agriculture and Agri-Food Canada, 107 Science Place, Saskatoon, SK S7N 0X2, Canada; yong-bi.fu@agr.gc.ca

**Keywords:** average pairwise dissimilarity, individual genomic distinctness, genomic variability, single nucleotide polymorphism, structural variant, plant germplasm characterization

## Abstract

The average pairwise dissimilarity (APD) between one plant sample and other assayed samples based on genetic markers was developed in 2006 to assess genetic distinctness and genetic redundancy in a plant germplasm collection. With the availability of abundant genomic variants across a genome, APD can be expanded to measure individual genomic distinctness. This study was conducted to assess the applicability of APD estimates in measuring the individual genomic distinctness of 1789 indica and 854 japonica rice samples based on published genome-wide single-nucleotide polymorphism (SNP) and structural variant (SV) data. It was found that the acquired APD estimates were weakly or not correlated between the SNP and SV data sets in the indica or japonica samples, respectively. For the indica samples, the APD estimates based on the SNP and SV data ranged from 0.1779 to 0.3277 and from 0.2297 to 0.4096, respectively. For the japonica samples, the SNP-based and SV-based APD estimates varied from 0.1774 to 0.3029 and from 0.1534 to 0.3459, respectively. These APD estimates were highly negatively correlated with the estimates of individual inbreeding coefficients and can identify the most genomically distinct rice germplasm that are compatible with those revealed through principal component analysis. Also, a reliable APD estimation was found to require 5000 to 10,000 random genomic SNPs or SVs. These findings together are significant, not only in demonstrating the informativeness of APD estimates in the identification of individuals with variable genomic distinctness, but also in providing guidance for APD applications to measure individual genomic distinctness.

## 1. Introduction

A genetic marker-based measure called an average pairwise dissimilarity (APD) was developed in 2006 to assess genetic distinctness and genetic redundancy in a plant germplasm collection for better germplasm management and conservation [1]. The APD measure is based on acquired genetic marker data to generate an APD value of one plant sample against the remaining assayed samples and provides a new means to characterize a unique genetic feature of plant samples [2], depending on the type of genetic markers, and to allow for the identification of genetically distinct or redundant samples. A higher APD value indicates that the sample is more genetically distinct than the other samples with lower APD values. Such an APD measure, however, differs from those measures of individual genetic diversity, such as allelic richness and rarity, heterozygosity [3,4], and inbreeding coefficient [5], as an APD value for a sample represents another unique feature of genetic variability relative to the other samples.

Applications of APD estimates to identify genetically distinct germplasm have been demonstrated to be instructive for the development of core subsets in a germplasm collection (e.g., [6]), for germplasm selection for safety backup in other facilities, and for broadening narrow genetic bases of breeding gene pools [7]. Assessing genetically redundant germplasm can help to identify and validate accession duplication [8,9,10,11]. The APD-based grouping of plant samples for genetic distinctness could also be more informative than classifications based on genetic structures inferred from STRUCTURE [12] or genetic associations revealed from a principal component analysis (or PCA; e.g., see [13]), thus facilitating the genetic categorization of conserved germplasm to enhance current and future germplasm uses [14]. Although the APD approach has been well cited in the scientific literature, it has not been applied as widely as hoped to assess genetic distinctness and the redundancy of conserved germplasm [15].

Individual genomic distinctness can be measured through the APD approach, given the availability or development of abundant genomic variants across a genome. This measurement represents a unique feature of genomic variation at the individual level and theoretically could contribute to many genomic applications by identifying genomically distinct individuals, beyond those applications mentioned above, to enhance plant germplasm conservation in genebanks. For example, plant germplasm with the highest individual genomic distinctness can be introduced into a breeding gene pool to broaden its genetic base [16], particularly for long-term breeding programs. Individual genomic distinctness could possibly be utilized to differentiate released varieties, particularly for outcrossing crops, for registration and breeder’s rights protection [17,18,19]. Identification of genomically distinct individuals could enhance the management and conservation of endangered species (e.g., see [20,21]). However, the applications of individual genomic distinctness have received little attention to date.

The APD approach has previously been applied to genomic single-nucleotide polymorphism (SNP) data [14], as published SNP data are available for many crops, but not to genomic structural variant (SV) data. SV data represents presence–absence genetic data and has only been available in the last several years due to the recent development of pangenomic analysis [22]. SVs consist of larger-scale genetic changes like insertions, deletions, duplications, and inversions that involve lengthy DNA sequences [23], while SNPs represent changes to a single DNA base pair. SVs also seem to impact more of the genome than single-nucleotide variations [24]. Thus, SNP and SV data may carry different types of genetic information across a genome [25].

The objective of this study was to assess the applicability of APD estimates to measure the individual genomic distinctness of rice (*Oryza sativa* L.) germplasm based on published genome-wide SNP [26] and SV [27] data. Specifically, SNP and SV data were utilized to obtain APD estimates for 1789 indica and 854 japonica rice samples. Correlations of SNP-based and SV-based APD estimates were analyzed and SNP-based and SV-based APD rankings of rice germplasm were compared. APD-based identifications of rice germplasm were also compared with association patterns revealed by the corresponding PCAs. Furthermore, the APD estimates were correlated with individual inbreeding coefficient estimates and the effects of variable numbers of random genomic variants on APD estimation were assessed. We hope this assessment will provide useful guidance for APD applications to measure individual genomic distinctness and stimulate more research in this area.

## 2. Materials and Methods

### 2.1. Acquisition of Published Rice Genomic Data

The SNP genotype data of 3K rice germplasm [26] was acquired on 26 September 2024 as a single rice VCF file called 3KriceRG404KcoreSNPdataset.vcf from the Rice SNP-Seek database (https://snp-seek.irri.org, accessed on 8 October 2025). The related passport data file (41586_2018_63_MOESM3_ESM.xlsx or Supplementary Data 3; [26]) was downloaded from https://www.nature.com/articles/s41586-018-0063-9#Sec34 (accessed on 1 December 2025). Similarly, the SV data of the 3K rice germplasm was acquired in PLINK format from Dr. Dmytro Chebotarov, the senior co-author of a paper on rice structural variants [27]. This SV data set has 311,518 SVs for 3024 rice samples that were filtered with a genotype missing rate of 0.2 or larger and an allelic frequency of 0.01 or lower (Dr. Dmytro Chebotarov, personal communication, 19 August 2025), and it differs from the original 3K RG 2.3mio biallelic indel data set in the Rice SNP-Seek Database. Since Dr. Chebotarov indicated that the SV calls across samples were clustered in case of significant reciprocal overlaps and the boundaries of SVs in each cluster may differ across samples, it is important to note that the alleles recorded in the filtered SV BIM file are only representative of the cluster and may not always coincide with the corresponding call at the same chromosomal position in the sample.

### 2.2. Data Processing

The acquired SNP and SV data sets were separated into indica and japonica group data sets to reflect two genetically recognized rice groups. Lists of rice subgroups were extracted from the passport data provided by Wang et al. [26]. The sample lists of the japonica and indica groups were used to extract the samples into their respective VCF files using BCFTools view [28] with the filtering of monomorphic sites only. This effort generated a VCF file for the japonica group data set with 854 samples and 308,053 loci and a VCF file for the indica group data set with 1789 samples and 387,115 loci. These SNP data sets were used by Fu [14] to generate APD values for rice samples. Similarly, the original SV PLINK files were split into two PLINK files (one for 1789 indica samples and one for 854 japonica samples), each with the same 311,518 SVs, before further filtering. The splitting was performed using PLINK v1.9.0-b.7.7 [29] with the new FAM files based on the lists of rice subgroups. As described below, APD estimation is dependent on the R package SNPRelate [30], which can handle large genomic data sets with a high computational efficiency, and SNPRelate requires the conversion of a VCF or PLINK file to GDS format. Thus, extra efforts were made to convert each SNP or SV data set into a GDS file, and four new data sets in GDS format were generated: indica-SNP, japonica-SNP, indica-SV, and japonica-SV.

### 2.3. APD Analysis

Each data set was first analyzed with respect to allelic frequency or minor allelic frequency and missing SNP data, allowing for a better understanding of the variability and heterogeneity of these data sets. For each data set, APD and its standard deviation were obtained for each sample using the published APD.r script [14] in an R v. 4.1.2 environment [31]. The R script was specifically written for an APD analysis following the method by Fu [1]. Briefly, in a typical marker-based characterization of self-fertile plant germplasm with *N* samples that are assayed at many SNP loci, a given sample can form *N* − 1 pairs with the remaining assayed samples. For each of such pairs, the genotypic similarity (*S*) can be calculated based on SNP genotypes following the simple matching coefficient of Sokal and Michener [32] and the pairwise dissimilarity is 1 − *S*. The average pairwise dissimilarity (or APD) for the given sample can be obtained by averaging all *N* − 1 pairwise dissimilarity values. The higher the APD value obtained for the given sample, the more genetically distinct the sample is. The lower the APD value, the more genetically redundant the sample is among the assayed samples.

The published APD.r script was modified specifically for each data input and the number of computational threads used for each data set, depending on the size of the data set. The modification also included the filtering of variants with a minor allelic frequency of 0.01 to remove extreme rare alleles (e.g., singleton) and applying a genotype missing threshold of 0.05 for SNPs [14] and 0.2 for SVs to ensure comparable numbers of genomic variants. The filtering generated much fewer SNP loci than those used by Fu [14]. For the computational analysis, a Conda [33] environment with R v. 4.1.2 was created on Agriculture and Agri-Food Canada’s Biocluster high-performance computing platform to run the R package, SNPRelate (v. 1.28.0; [30]), and its dependencies. For each data set, the analysis lasted up to 3 h with 16 threads. The generated APD estimates (and their standard deviations) are listed in Table S1 for the indica group and Table S2 for the japonica group.

### 2.4. Associating APD Estimates with Other Genetic Estimates

PCA is commonly used to assess the genetic relationships of assayed samples based on a given set of molecular data. PCA plots usually show the genetic relationships of assayed samples, allowing for the identification of genetically distinct samples based on clustering patterns. To compare the associations identified by APD estimates with those revealed by PCA, a PCA plot was generated for each data set using the snpgdsPCA() function in SNPRelate. The PCA plots were regenerated in R using the plot() function, highlighting samples with the top 10%, 20%, and 30% APD estimates (Figures S3 and S4). The percentage of the convex hull area for those samples, determined by a given level of APD estimates over the convex hull area of the entire PCA sample space, was calculated with a custom R script utilizing the base R function chull() and R package sp function Polygon() [34]. The convex hull area percentage (HAP) allows for the quantification of the PCA sample spaces shared by APD-identified samples. The higher the HAP, the greater the shared PCA sample spaces.

Individual inbreeding coefficient (IBC; [5]) for each rice sample was also estimated in the four data sets using the SNPRelate snpgdsIndInb() function. These IBC estimates were correlated with the corresponding APD estimates using a linear regression (or R lm() function), and the correlation was plotted using R plot() function for each data set. These correlations will allow for the assessment of the theoretical expectation that the samples with higher IBC estimates would have lower genomic variations and thus lower APD estimates, as APD estimates represent one unique feature of genomic variability.

### 2.5. Impact of Variant Number on APD Estimation

Previous SNP-based analyses of other crops [14] suggested that 5000 to 10,000 genome-wide SNPs are generally required for an effective APD analysis, but little is known if the suggestion still holds for rice germplasm, particularly based on SV data. Effort was made in this analysis to evaluate the effects of variant number on APD estimates. Specifically, for each data set, the numbers of genomic variants were set as 1000, 3000, 5000, 10,000, and 15,000. For each given number of variants, a data set was generated by randomly selecting the given number of variants across the genome to estimate APD and the Pearson correlation coefficient (r) was calculated between the estimated APD with the given number of variants and those with all variants of the original data set. This procedure was repeated 10 times and the mean and standard deviation of the r values were generated for a given number of random variants of each data set. A custom shell script was written to perform the analysis and the selection of random variants was performed using the snpgdsSelectSNP() function from the SNPRelate package.

## 3. Results

### 3.1. Variability of APD Estimates in Four Data Sets

After filtering for minor and/or allelic frequency and missing genotypes, the four data sets had genomic variants ranging from 135,493 to 261,475, which were widely distributed across 12 chromosomes (Figure S1). The lowest numbers of variants were present in chromosome 9 with 8665 SNPs and 6004 SVs. Minor and/or allelic frequencies for these variants followed an L-shape distribution in each data set (Figure S2), a pattern typically expected for both SNP and SV data.

**Figure 1:**
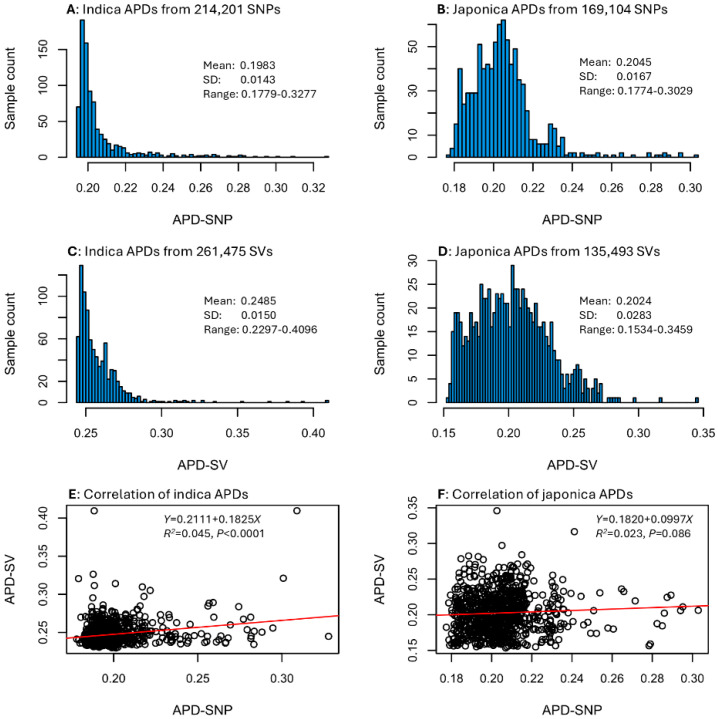
Distributions of APD estimates for the four data sets (**A**–**D**) and correlations of SNP-based and SV-based APD estimates for 1789 indica (**E**) and 855 japonica (**F**) samples.

An APD estimate was calculated for every sample in each data set (see Tables S1 and S2). The APD estimates displayed different frequency distributions between the two sample groups, but similar frequency distributions between SNP and SV data types for each rice group (Figure 1). For example, the 1789 indica samples showed L-shape distributions of APD estimates based on SNPs and SVs, while the 855 japonica samples exhibited skewed normal distributions of SNP-based and SV-based APD estimates. Specifically, APD estimates for the indica group ranged from 0.1779 to 0.3277 with a mean of 0.1983 for the SNP data (Figure 1A) and from 0.2297 to 0.4096 with a mean of 0.2485 for the SV data (Figure 1C). The APD estimates for the japonica group ranged from 0.1774 to 0.3029 with a mean of 0.2045 for the SNP data (Figure 1B) and from 0.1534 to 0.3459 with a mean of 0.2024 for the SV data (Figure 1D). However, the correlation between indica SNP-based and SV-based APD estimates was statistically significant with a weak correlation (or *R*^2^ = 0.045; Figure 1E), while the correlation between japonica SNP-based and SV-based APD estimates was not statistically significant at *p* < 0.05 (Figure 1F). These correlations suggested that the assayed rice samples had different sets of APD estimates for the SNP and SV data. Thus, the identification of the most genomically distinct rice germplasm that is based on APD estimates of the SNP or SV data sets can differ. For a given set of APD estimates, the higher the APD estimates, the more genomically distinct the samples are. The identification can be performed with the provided APD estimates shown in Tables S1 and S2.

To illustrate the APD ranking, Table 1 shows the rankings of 25 indica or japonica rice samples with the highest APD estimates in each data set. The three most genomically distinct indica samples were CX401, BO39, and B221 from three countries for the SNP data set and BO39, CX101, and IRIS_313-11746 from two countries for the SV data set. Similarly, the three most genomically distinct japonica samples were IRIS_313-10459, IRIS_313-10444, and IRIS_313-10872 from three countries for the SNP data set and IRIS_313-7992, IRIS_313-8027, and IRIS_313-8637 from three countries for the SV data set. Overall, these 25 top-ranked indica or japonica rice samples originated from 10 (for indica-SV) and 11 (for indica-SNP) to 13 countries (for japonica-SNP and japonica-SV). Thus, the genomic distinctness was apparently associated with the sample origin. However, among the 25 indica or japonica samples from the SNP and SV data sets, only two (BO39 and B221) or one (IRIS_313-8027), respectively, were shared, suggesting that SNP- and SV-based APD rankings differed.

An effort was also made to calculate the sample counts shared between the two sets of rice samples ranked separately in a given percentage of SNP-based or SV-based APD estimates, as shown in Table 2. For the 1789 indica samples, 18 samples were ranked in the top 1% based on SNP-based and also SV-based APD estimates, but only 2 samples were shared between the two sets of 18 samples, representing 11.1% of the top-ranked samples. Similarly for the 854 japonica samples, there were 256 (out of 854) samples for both sets ranked in the top 30%, but there were only 89 samples present in both sets, representing 34.8% of the samples ranked in the top 30%. These results indicate that the identification of the most genomically distinct rice germplasm could differ as SNP-based and SV-based APD estimates have different sample rankings. This is expected, given the findings of weak correlations between SNP-based and SV-based APD estimates (Figure 1E,F).

### 3.2. Associations Between APD Estimates and Other Genetic Estimates

**Figure 2:**
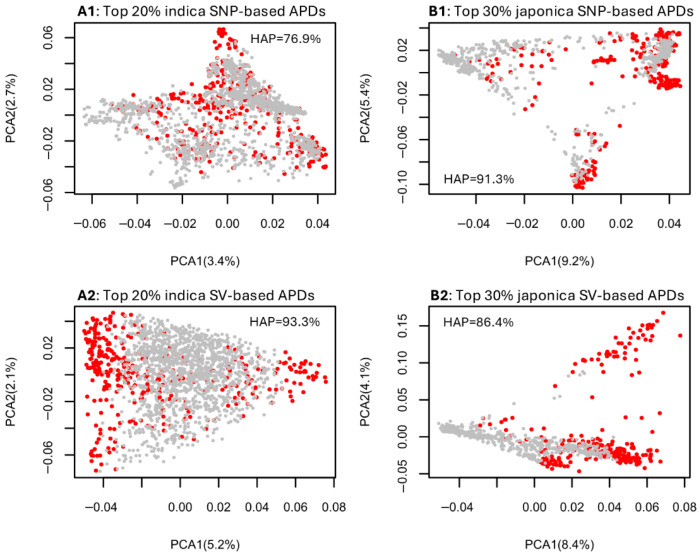
Illustrative PCA plots showing the genetic associations of the assayed rice groups based on the four data sets ((**A1**) for indica–SNP; (**B1**) for japonica–SNP; (**A2**) for indica–SV; (**B2**) for japonica–SV) and the identification in red of the most genomically distinct rice germplasm based on the highest 20% indica APD estimates (**A1**,**A2**) and the highest 30% japonica APD estimates (**B1**,**B2**). The hull area percentage (HAP) to quantify the PCA sample space shared by APD-identified samples is also given in each panel.

The PCA plots for the indica group, based on the SNP and SV data sets (Figure S3), revealed the genetic associations among assayed indica samples and highlighted the most genomically distinct rice germplasm corresponding to the top 10% (Figure S3(A1,B1)), 20% (Figure S3(A2,B2)), and 30% (Figure S3(A3,B3)) of APD estimates. For the indica samples, the convex hull area percentages of the SNP-inferred PCA sample space shared by the samples with the top 10% to 30% of APD estimates ranged from 68.1% to 79.2%, while those of the SV-inferred PCA sample space ranged from 87.8% to 94.3%. Similarly, the PCA plots for the japonica group (Figure S4) illustrated the most genomically distinct rice germplasm based on the top 10% (Figure S4(A1,B1)), 20% (Figure S4(A2,B2)), and 30% (Figure S4(A3,B3)) of APD estimates. The convex hull area percentages of the SNP-inferred PCA sample space shared by the japonica samples with the top 10% to 30% of APD estimates ranged from 59.0% to 91.3%, while those of the SV-inferred PCA sample space ranged from 77.2% to 86.4%. Summarized plots in Figure 2 demonstrate that the indica samples identified with the highest 20% APD estimates and the japonica samples identified with the highest 30% APD estimates essentially covered their respective PCA sample spaces, with the convex hull area percentages ranging from 76.9% to 93.3%. These results indicate that the APD-based identification of individuals with the most genomic distinctness would yield the same or similar results to the association patterns observed in a PCA plot.

**Figure 3:**
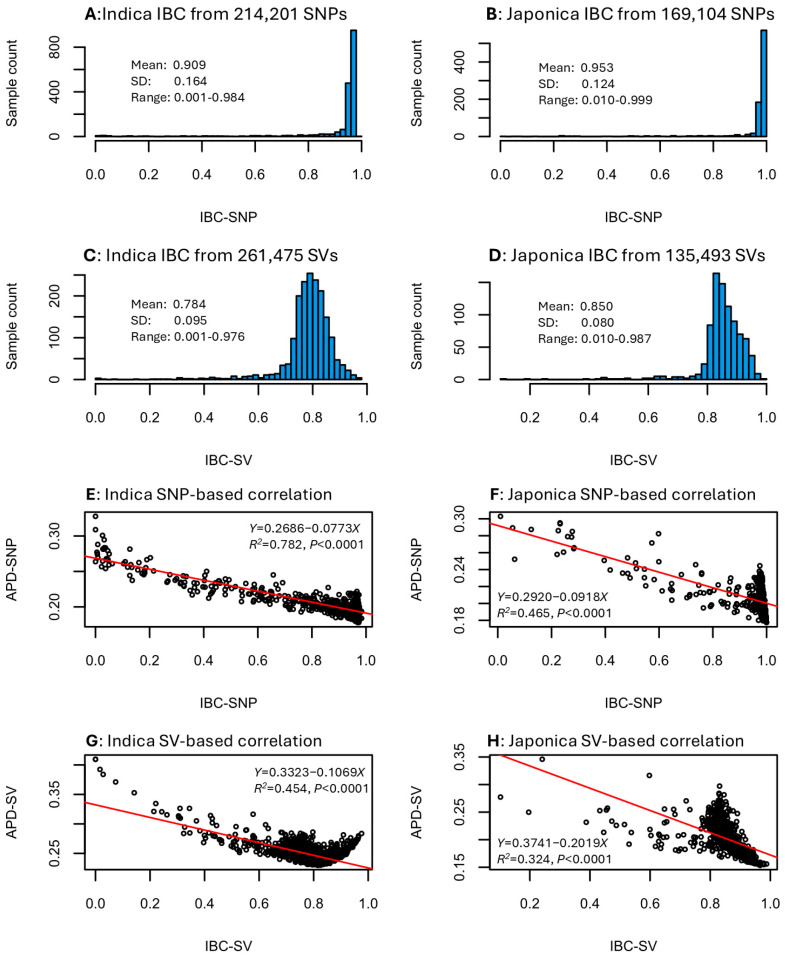
Distributions of the individual inbreeding coefficient (IBC) estimates in the four data sets (**A**–**D**) and correlations between IBC and APD estimates (**E**–**H**) with respect to rice group and variant data set.

The IBC estimates for the indica and japonica samples were obtained based on the SNP and SV data sets. These estimates followed left L-shaped distributions for the SNP data (Figure 3A,B) and skewed normal distributions for the SV data (Figure 3C,D). Specifically, the IBC estimates for the indica group ranged from 0.001 to 0.984 with a mean of 0.909 for the SNP data (Figure 3A) and from 0.001 to 0.976 with a mean of 0.784 for the SV data (Figure 3C). The IBC estimates for the japonica group ranged from 0.010 to 0.999 with a mean of 0.953 for the SNP data (Figure 3B) and from 0.010 to 0.987 with a mean of 0.850 for the SV data (Figure 3D). Evaluating the correlations between IBC and APD estimates in the four data sets revealed a highly significant negative correlation in each data set (Figure 3E–H). The significant correlation coefficients were −0.884 for indica SNP data (Figure 3E), −0.682 for japonica SNP data (Figure 3F), −0.674 for indica SV data (Figure 3G), and −0.569 for japonica SV data (Figure 3H). Thus, an individual sample with a higher inbreeding coefficient estimate would have a lower genomic distinctness. The negative correlation for the SNP data seemed to be larger than that for the SV data. The findings of these negative correlations confirmed the expectation that higher inbred individuals are known to be less genomically diverse.

### 3.3. The Effects of Variant Numbers on APD Estimation

The correlations between APD estimates with all genomic variants and with a range of smaller numbers of genomic variants from 1000, 3000, 5000, 10,000, and 15,000 were obtained with 10 replications in each of the four data sets. Figure S5 illustrates the results of one run of the correlations between APD estimates using all genomic variants and a given number of random variants in each data set. The results revealed that an effective APD estimation required 5000 to 10,000 random genomic SNP or SVs, if an acceptable threshold of correlation coefficient estimate is set to be 0.98. Summarizing the correlations over 10 replications confirmed the results of the single run with the coefficient of variation of 0.0022 or smaller when the number of random genomic variants was 5000 or larger (Table 3).

## 4. Discussion

This assessment represented the first APD application with SV data and revealed several interesting findings for the identification of the most genomically distinct rice germplasm based on APD estimates. First, APD estimates of the rice germplasm differed between the SNP and SV data sets and showed weak or no correlations between the two data sets (Figure 1). Second, the APD-based identification of the most genomically distinct rice germplasm was compatible with those revealed by PCA (Figure 2). Third, APD estimates were highly negatively correlated with the estimates of individual inbreeding coefficients (Figure 3). Fourth, a reliable APD estimation required 5000 to 10,000 genomic SNPs or SVs (Table 3). These findings are significant, not only in demonstrating the informativeness of APD estimates in the identification of individuals with variable genomic distinctness, but also in providing some useful guidance for APD applications to measure individual genomic distinctness.

The observation of weak or no correlations between SNP-based and SV-based APD estimates is novel and interesting. Such weak correlations may have reflected the effects of SV genotyping errors from clustering for significant reciprocal overlaps and differing boundaries of SVs in each cluster across samples [27]. However, it is also possible that SNP and SV data may truly represent different types of genetic variation present across a genome. Plant SV data is not fully understood [22,24,25,35], not only for this distinctness assessment but also for applications such as genome-wide association studies of phenotypic traits (e.g., see [36]). SV data is relatively new as genetic presence–absence data and has been generated only from pangenome analyses over the last decade [25]. Interestingly, the rice SV data showed a chromosomal distribution pattern (Figure S1(C,D)) and L-shaped allelic frequency distribution (Figure S2(C,D)) similar to those of the rice SNP data (Figures S1(A,B) and S2(A,B)). Thus, more research is still needed to understand the new genetic data type of genomic structural variants.

The finding of strong negative correlations (r = −0.569 to −0.884) between APD estimates and individual inbreeding coefficient estimates (Figure 3E–H) is noteworthy but genetically expected. Individual samples with higher inbreeding coefficient estimates are expected to have lower genetic variability and their genomic distinctness is expected to be lower, as APD estimates measure the unique feature of genomic variability. The interesting features of this finding are the strength of the correlations and the slightly weaker correlations in the SV data (i.e., −0.569 to −0.674) than the SNP data (i.e., −0.682 to −0.884). This genomic feature suggested that the SV data represents a different type of genomic variability than the SNP data [24]. It is possible that the rice SV data contained more non-genetic (or non-Mendelian) variability than the SNP data or the weaker correlation is simply due to the low informativeness of presence–absence data.

The analysis of the informativeness of the APD-based identification of individual genomic distinctness in a PCA plot space was useful, as it demonstrated that the indica samples with the top 20% highest APD estimates and the japonica samples with the top 30% APD estimates can be identified to cover most of a corresponding PCA sample space with the shared convex hull area percentages of 76.9% to 93.3% (Figure 2, Figures S3 and S4). Simply put, the genomic variability present in the indica or japonica samples can be identified by the highest 20% or 30% of APD estimates. Thus, APD estimates were not only genetically informative for the identification of individual genomic distinctness, but also provided a quantitative measure of individual genomic distinctness, showing some advantage over PCA. Interestingly, the SV-based APD estimates seemed to have better coverage of the corresponding PCA plot space than the SNP-based APD estimates, suggesting that SV data is more informative for identifying individual genomic distinctness than SNP data.

This assessment also provided insights into the number of genomic variants required for an effective APD application. The finding that a reliable APD analysis generally requires 5000 to 10,000 genome-wide SNPs or SVs (Figure 3) confirms previous findings based on genomic SNPs from other crop plants such as barley, wheat, and chickpea [14]. Furthermore, 3000 to 5000 random genomic SVs can also achieve a better accuracy of APD estimation than 3000 to 5000 random genomic SNPs (Figure S5). Such numbers of genomic SNP or SV data points are attainable in many genomic applications, even with lower coverage genotyping applications such as genotyping-by-sequencing [37,38], making reliable APD analysis feasible. Also, an extra effort was made to correlate SNP-based APD estimates from this assessment and those of Fu [14] with much more SNP loci and revealed a correlation coefficient of 0.938 for indica samples and of 0.900 for japonica samples. Thus, filtering SNP loci with missing values of 20% or higher and a minor allelic frequency of 0.01 or smaller generated fewer SNP loci for this assessment, but it did not significantly affect the APD estimations. These findings, along with previous findings on missing data, allelic frequency, and data type [14,39], will provide effective guidance for improved APD applications.

Together, this assessment not only demonstrated that individual genomic distinctness of rice germplasm can be measured with an average pairwise dissimilarity of genomic SNP and SV data, but also provided supplemental guidance for improved APD applications, particularly with SV data. For example, APD estimations and the identification of the most genomically distinct germplasm should be performed separately on SNP and SV data, as SNP-based and SV-based APD estimates differ, as shown in Figure 1. As demonstrated in Figure 3, APD estimates based on genomic variants are correlated negatively with inbreeding coefficients, thus confirming the informativeness of APD estimates in measuring the unique feature of genetic variability across the genome at the individual level. APD estimation can also be as informative as PCA to identify individuals with the highest genomic distinctness, but has the advantage over PCA in providing a quantitative measure of individual genomic distinctness.

With the growing availability of genome-wide variant data, APD estimation could play more roles in genomic applications, besides identifying individual genomic distinctness for plant germplasm conservation and widening the plant breeding gene pool. For example, diagnostic tools based on APD estimates could aid in the identification of plant varieties for registration and breeder’s rights protection [17,18,19]. APD estimation can enhance the development of effective strategies for maintaining genetic diversity in endangered plant species through measuring individual genomic distinctness and identifying effective conservation targets (e.g., see [20,21]). Similarly, APD estimates can be acquired as a supplemental measure to individual inbreeding coefficient estimates to inform conservation managements for other endangered organisms, such as the endangered northern quoll (*Dasyurus hallucatus* Gould) [40] and North Atlantic right whales (*Eubalaena glacialis* Müller) [41]. These potential applications highlight the value of APD estimation as a genomic tool for advancing genetic research and enhancing practical conservation efforts.

## 5. Concluding Remarks

This assessment revealed several significant findings for the identification of the most genomically distinct plant germplasm based on APD estimates. APD estimates of rice germplasm differed between the SNP and SV data sets and showed weak or no correlations between the two data sets. The APD-based identification of rice germplasm with the most genomic distinctness was compatible with those revealed by PCA. APD estimates were highly negatively correlated with the estimates of individual inbreeding coefficients. Also, a reliable APD estimation required 5000 to 10,000 genomic SNPs or SVs. These findings together are significant, not only demonstrating the informativeness of APD estimates in the identification of individuals with variable genomic distinctness but also providing guidance for improved APD applications to measure individual genomic distinctness.

## Figures and Tables

**Table 1:** The rankings of 25 indica or japonica rice samples with the highest APD estimates in each of the four data sets. The description for genetic stock shows only the first pedigree with “::” used to save space—more information can be found in the Supplementary Excel files (Tables S1 and S2). SD is standard deviation.

Sample.ID	Genetic Stock	Origin	APD	SD	Sample.ID	Genetic Stock	Origin	APD	SD
indica-SNP					japonica-SNP				
CX401	TOG 7291	Burkina Faso	0.328	0.011	IRIS_313-10459	PI 160862-1::	China	0.303	0.021
B039	IRAT 10	Cote d’Ivoire	0.309	0.015	IRIS_313-10444	Fortuna colorado::	Central America	0.295	0.025
B221	Fanhaopi	China	0.301	0.013	IRIS_313-10872	ARC 11802::	India	0.294	0.021
IRIS_313-11269	ARC 14632::	India	0.295	0.007	CX307	HP 121	China	0.289	0.033
IRIS_313-11260	ARC 13591::	India	0.288	0.009	CX243	IR 47686-6-2-1-1	Philippines	0.287	0.029
CX542	RR2-6	China	0.283	0.013	IRIS_313-10771	GOGO RAJAPAN::	Indonesia	0.286	0.017
IRIS_313-11176	SXC 216::	India	0.283	0.010	CX367	Haogelao	China	0.285	0.025
IRIS_313-11242	OR 117-8::	India	0.282	0.011	IRIS_313-8872	571::	Thailand	0.282	0.021
IRIS_313-10787	KWATIK PUTIH::	Indonesia	0.281	0.011	IRIS_313-11380	CA ONG (WHITE)::	Philippines	0.279	0.023
IRIS_313-10705	Padi pulot melayang::	Malaysia	0.281	0.006	IRIS_313-11008	PULUT CENRANA::	Indonesia	0.278	0.028
CX4	93072	China	0.279	0.013	IRIS_313-10841	KETAN NANGKA::	Indonesia	0.272	0.035
IRIS_313-10825	KEMA 5::	Sierra Leone	0.277	0.010	CX106	SAL BUI BAO	Viet Nam	0.266	0.038
IRIS_313-8935	ARC 18061::	India	0.275	0.015	CX282	Lijiangxintuanheigu	China	0.265	0.025
B247	Jinnante B	China	0.274	0.013	IRIS_313-8137	SAGRES::	Portugal	0.261	0.034
IRIS_313-11098	KOLUBA::	Sierra Leone	0.269	0.018	B047	Zhenfu 8	South Korea	0.258	0.046
IRIS_313-11817	KHAOSAING::	Myanmar	0.269	0.010	IRIS_313-11329	PADI JALAI BELA::	Malaysia	0.254	0.032
CX13	R644	China	0.268	0.016	IRIS_313-8127	POLIZESTI 28::	Bulgaria	0.252	0.029
IRIS_313-11848	PULUT BURUNG::	Malaysia	0.267	0.015	CX284	Han 502	China	0.251	0.028
IRIS_313-11095	MAK EA NAM::	Laos	0.267	0.011	IRIS_313-8323	REXARK ROGUE::	USA	0.249	0.036
CX69	MR 167	Malaysia	0.266	0.011	CX11	Gumei 2	China	0.248	0.023
IRIS_313-9190	CODE NO 31323::	India	0.265	0.010	IRIS_313-10766	DJALAWARA::	Indonesia	0.246	0.035
IRIS_313-9114	LEUANG 28-1-87::	Thailand	0.264	0.009	IRIS_313-10816	SIDJERO GUNDIL::	Indonesia	0.244	0.037
IRIS_313-10690	LARONDJAWI::	Indonesia	0.264	0.014	IRIS_313-8027	ANSEATICO::	Italy	0.241	0.028
IRIS_313-10484	Pilit(7480)Sel(ci12007)::	Philippines	0.263	0.017	CX77	Lemont	USA	0.241	0.029
IRIS_313-11132	KALALAN::	Myanmar	0.261	0.012	IRIS_313-8032	PIEMONTE::	Italy	0.239	0.037
indica-SV					japonica-SV				
B039	IRAT 10	Cote d’Ivoire	0.410	0.021	IRIS_313-7992	VARY LAVA 90::	Madagascar	0.346	0.019
CX101	Hei Mi Chan	China	0.409	0.024	IRIS_313-8027	ANSEATICO::	Italy	0.317	0.028
IRIS_313-11746	E 2070::	China	0.392	0.012	IRIS_313-8637	BALINGMI::	Bhutan	0.297	0.031
IRIS_313-11901	TI NGI::	Thailand	0.384	0.009	IRIS_313-15907	INIA TACUARI::	Uruguay	0.284	0.030
IRIS_313-11523	ADIALLO::	Senegal	0.371	0.019	IRIS_313-8096	SR 113::	Spain	0.283	0.025
IRIS_313-11307	ARC 15387::	India	0.353	0.025	IRIS_313-10059	DACHEONGBYEO::	South Korea	0.282	0.023
IRIS_313-11889	MURGI BRINJ::	Pakistan	0.334	0.018	IRIS_313-10840	YE ZO::	South Korea	0.278	0.028
CX75	At 354	Sri Lanka	0.326	0.020	CX351	053 A-3	China	0.277	0.042
IRIS_313-11750	L 10595::	China	0.326	0.030	IRIS_313-8090	MARENY::	Spain	0.271	0.021
B221	Fanhaopi	China	0.321	0.016	IRIS_313-10080	Hirakawa okute::	Japan	0.271	0.026
CX97	Budda	India	0.321	0.013	IRIS_313-10075	SANGOKU::	Japan	0.271	0.031
IRIS_313-8450	498-2A BR 8::	India	0.316	0.023	IRIS_313-8112	CHIPKA::	Bulgaria	0.270	0.039
IRIS_313-8591	SIAM ER 32::	Malaysia	0.315	0.029	IRIS_313-12348	LOUK NOK::	Laos	0.269	0.027
CX118	Yetuozai	China	0.314	0.037	IRIS_313-15910	CYPRESS::	USA	0.269	0.025
IRIS_313-11869	MA WEI ZHAN::	China	0.313	0.026	IRIS_313-12262	NYAE::	Laos	0.267	0.028
CX18	Zaoxian 14	China	0.312	0.028	IRIS_313-8039	LOTO::	Italy	0.267	0.028
IRIS_313-10671	ARC 10581::	India	0.310	0.014	IRIS_313-12337	DOK HIEN NOI::	Laos	0.267	0.029
IRIS_313-11911	YUN NAN ZHAN::	China	0.309	0.020	IRIS_313-8076	PELDE::	Australia	0.267	0.022
B013	Sri Lanka 1	Sri Lanka	0.305	0.016	IRIS_313-12352	MEE::	Laos	0.266	0.030
IRIS_313-11423	C 1016-1::	Philippines	0.302	0.021	IRIS_313-12350	Mak kheua kang::	Laos	0.266	0.029
IRIS_313-11471	CULALANSI::	Philippines	0.299	0.041	IRIS_313-10776	KABADOKA::	Indonesia	0.264	0.026
B012	2037 (Rajahamsal)	India	0.297	0.017	IRIS_313-15904	JINBUBYEO::	South Korea	0.264	0.025
CX392	SARD	_no_info	0.295	0.030	IRIS_313-12254	BAN BONG::	Laos	0.262	0.026
IRIS_313-11558	NULI::	Bangladesh	0.295	0.020	IRIS_313-12332	DENG NYAY::	Laos	0.261	0.025
IRIS_313-11887	IR 19058-107-1::	Philippines	0.292	0.010	IRIS_313-8095	SHSS 53::	Spain	0.261	0.032

**Table 2:** The shared top ranking of rice germplasm based on the SNP-based and SV-based APD estimates. Shared sample count is the number of samples that were present in two sets of samples ranked separately in a given percentage of the SNP-based or SV-based APD estimates.

Top Rank (%)	SNP-Based APD Sample Count	SV-Based APD Sample Count	Shared Samples Count	Shared Sample Count (%)
1789 indica samples				
1	18	18	2	11.1
5	89	89	9	10.1
10	179	179	29	16.2
15	268	268	58	21.6
20	358	358	86	24.0
25	447	447	130	29.1
30	537	537	187	34.8
854 japonica samples				
1	9	9	0	0.0
5	43	43	1	2.3
10	85	85	5	5.9
15	128	128	19	14.8
20	171	171	44	25.7
25	214	214	68	31.8
30	256	256	89	34.8

**Table 3:** Statistics of the Pearson correlation coefficient estimates between the APD estimates using all genomic variants and a given number of random variants for the SNP and SV data sets of indica and japonica rice groups. The results for 5000 and 10,000 random variants are highlighted in bold.

The Number of Random Variants	SNP			SV		
	Mean	Standard Deviation	Coefficient of Variation	Mean	Standard Deviation	Coefficient of Variation
1789 indica lines						
1000	0.91683	0.00797	0.00870	0.92369	0.00719	0.00779
3000	0.97079	0.00314	0.00324	0.97177	0.00292	0.00300
**5000**	**0.98299**	**0.00096**	**0.00098**	**0.98419**	**0.00084**	**0.00086**
**10,000**	**0.99085**	**0.00066**	**0.00067**	**0.99179**	**0.00094**	**0.00095**
15,000	0.99414	0.00066	0.00067	0.99484	0.00038	0.00038
854 japonica lines						
1000	0.94226	0.00923	0.00979	0.97989	0.00239	0.00244
3000	0.97761	0.00496	0.00507	0.99313	0.00083	0.00083
**5000**	**0.98721**	**0.00213**	**0.00216**	**0.99597**	**0.00051**	**0.00052**
**10,000**	**0.99265**	**0.00145**	**0.00146**	**0.99780**	**0.00022**	**0.00022**
15,000	0.99548	0.00100	0.00101	0.99860	0.00023	0.00023

## Data Availability

The data presented in this study are available in the Supplementary Materials.

## Supplementary Materials

The following supporting information can be downloaded at https://doi.org/10.6084/m9.figshare.30573032 (accessed 1 December 2025): two supplemental tables (Tables S1 and S2) in Excel files and five supplemental figures (Figures S1–S5) in a PDF file.
